# Histological Analysis of Root Surface Treatment with Tetracycline and Ampicillin in the Repair Process of Rat Teeth Subjected to Delayed Replantation

**DOI:** 10.3390/jcm14134443

**Published:** 2025-06-23

**Authors:** Guilherme Assumpção Silva, Celso Koogi Sonoda, Marina Fuzette Amaral, Vitor Hugo Gonçalves Sampaio, Daniela Atili Brandini

**Affiliations:** Department of Diagnosis and Surgery, School of Dentistry, São Paulo State University (Unesp), Araçatuba, Street José Bonifácio Nº 1193, Araçatuba 16015-050, SP, Brazil; guilherme.assumpcao@unesp.br (G.A.S.); celso.k.sonoda@unesp.br (C.K.S.); marina_fuzette.amaral@hotmail.com (M.F.A.); vitor.sampaio@unesp.br (V.H.G.S.)

**Keywords:** dental avulsion, root resorption, periodontal ligament, ampicillin, tetracycline

## Abstract

**Background/Objectives:** Avulsion is a major cause of tooth loss, and its treatment involves replantation. This study analyzed the repair process of incisive teeth subjected to delayed replantation after topical treatment with ampicillin and tetracycline. **Methods:** Forty male rats were equally divided into four groups: immediate replantation (IM), ampicillin (AM), tetracycline (TR), and dry medium (SE). In the IM group, replantation was performed 5 min after experimental avulsion, whereas in the SE group, the teeth were kept in a dry medium for 60 min. In the AM and TR groups, the teeth were stored in whole bovine milk for 60 min, and then immersed in their respective ampicillin and tetracycline solutions for 5 min. Euthanasia was performed 60 days after the experimental surgery. Histological slides were stained with Hematoxylin and Eosin for quantitative and qualitative analyses. **Results**: No statistically significant differences were observed among the IM, AM, and TR groups regarding the total resorption area, reattached periodontal ligament fibers, and ankylosis. However, when compared to the SE group, these groups exhibited a smaller total resorption area (IM: *p* = 0.005; AM: *p* = 0.0007; TR: *p* = 0.03), a larger area of periodontal ligament fibers reattachment (IM: *p* = 0.0002; AM: *p* = 0.0002; TR: *p* = 0.02), and a lower presence of ankylosis (IM: *p* = 0.005; AM: *p* = 0.0002; TR: *p* = 0.03). The AM group exhibited the smallest inflammatory resorption. **Conclusions:** It is concluded that, in an aseptic environment, the use of topical antibiotics such as ampicillin and tetracycline in the treatment of the root surface during replantation of teeth stored in milk is beneficial to the periodontal repair process and root resorption. Notably, ampicillin demonstrated superior efficacy in mitigating inflammatory root resorption and better periodontal ligament reattachment.

## 1. Introduction

Dental avulsion is characterized by the complete displacement of a tooth from its socket, and is a significant cause of tooth loss, alongside caries and periodontal disease. The primary treatment is dental replantation. When performed promptly or after the avulsed tooth is preserved under conditions that maintain the vitality of the periodontal ligament and cementum layer, the chances of success increase.

One of the main factors for the survival of avulsed teeth is periodontal repair [1]. Damaged cells of the periodontal ligament reduce the resilience of the root and the surrounding alveolar bone. Furthermore, a damaged periodontal ligament no longer functions as a barrier between the dental tissue and the adjacent bone, leaving the root unprotected from active clastic cells, which results in root resorption [2].

Despite the importance of immediate replantation, clinical experience shows that this type of therapy rarely occurs, due to factors associated with injuries that pose a risk to the patient’s life, the presence of damage to the recipient site of the tooth to be replanted, and a lack of knowledge among the population and professionals about immediate replantation [3,4,5].

Delayed replantation is associated with a poor prognosis. To reduce the likelihood of root resorption, the tooth should be stored in an appropriate preservation medium to protect the viability of the remaining periodontal ligament cells. This will inhibit osteoclastic activity and inflammation caused by contamination of the root and root canals [6,7,8], activities that are related to periodontal repair and tooth survival.

The use of systemic antibiotic therapy as part of the replantation protocol is still controversial [9]. However, the International Association of Dental Traumatology (IADT) recommends the use of topical antibiotics to help slow down the process of resorption [10]. Studies evaluating the effects of amoxicillin and tetracycline have shown that the medicated groups exhibited better repair than the control groups [7,9]. However, there is still a need for additional studies to further clarify the effect of these medications on dental replantation. Given the above, based on the favorable tissue repair properties of these medications and the reduced bioavailability of tetracycline when administered orally, we considered it appropriate to histologically evaluate the topical use of ampicillin and tetracycline in the root treatment of avulsed teeth subjected to delayed replantation.

### Objective

This study analyzed the repair process of incisor teeth subjected to delayed replantation after topical treatment with ampicillin and tetracycline.

## 2. Material and Methods

### 2.1. Animals

Following approval by the Animal Use Ethics Committee (CEUA) of the School of Dentistry of Araçatuba—UNESP (No. 255-2022) on 30 November 2022, forty male Wistar rats (Rattus norvegicus albinus), weighing approximately 250 g and aged 120 days, were selected. The rats were housed in collective cages, five animals per cage, in a climate-controlled environment with a temperature maintained between 22 °C and 24 °C, under a controlled light cycle (12 h light/12 h dark). Healthy animals with no dental or periodontal alterations were obtained from the Animal Facility of the School of Dentistry of Araçatuba—UNESP. The animals were monitored before and throughout the entire experiment by a specialized professional (the institution’s veterinary physician). They were fed with ground solid feed and had access to water ad libitum. The sample size was calculated based on preliminary data from a pilot study, using a significance level of 5% and a beta power of 90%. A total sample size of 10 animals per group was determined. Sample size calculation was performed using the website Clincalc.com (ClinCalc LLC, Arlington Heights, IL, USA).

### 2.2. Dental Replantation

Prior to the surgical procedure, the animals were anesthetized via intramuscular injection with Xylazine Hydrochloride (Anasedan Agribrands of Brazil Ltd., Paulínia, SP, Brazil) at a dosage of 10 mg/kg of body weight for muscle relaxation, and Ketamine Hydrochloride (Agribrands of Brazil Ltd., Paulínia, SP, Brazil) at a dosage of 70 mg/kg of body weight for a sedative effect. For the surgical procedure, antisepsis of the anterior maxillary region was performed using povidone-iodine (Riodeine—Ind. Farmac. Rioquímica Ltda., São José do Rio Preto, São Paulo, Brazil), followed by syndesmotomy, luxation, and extraction of the upper-right incisor (URI) with the aid of adapted instruments. The extracted teeth were randomly selected (by lottery) and divided into four groups. In the Immediate replanted group (IM), the teeth underwent to extration, endodontic treatment and replantion (positive control) (Table 1). In the AM (ampicillin) group, after extraction, the teeth were immersed in pasteurized whole bovine milk (Parmalat Brasil S.A. Indústria de Alimentos, São Paulo, São Paulo, Brazil) for 60 min, followed by immersion for an additional 5 min in a suspension of 1 mg of ampicillin (Eurofarma Laboratórios S.A., Itapevi, São Paulo, Brazil) in 20 mL of saline solution (Fresenius Kabi, Barueri, São Paulo, Brazil). After extraction, in the TR (tetracycline) group, the teeth were immersed in pasteurized whole bovine milk for 60 min, followed by immersion for an additional 5 min in a suspension of 1 mg of tetracycline (Doxycycline hydrochloride—EMS—São Bernardo do Campo/SP, Brazil) in 20 mL of saline solution; and also in the SE (dry medium) group the teeth were kept in a dry environment on a bench for 60 min, followed by immersion in saline solution for 5 min (negative control) (Table 2). For endodontic treatment, the extracted teeth had their pulp removed retrogradely with the aid of a Kerr file #25 (SybronEndo Corporation, Orange, California, United States). The canals were then irrigated with saline solution and dried with absorbent paper points (MK Life Indústria e Comércio de Produtos Odontológicos Ltda., Curitiba, PR, Brasil). Subsequently, they were filled with a mixture of calcium hydroxide paste (Biodinâmica Química e Farmacêutica Ltda., Ibiporã, Paraná, Brazil) and propylene glycol Dinâmica Química Contemporânea Ltda., São Paulo, São Paulo), prepared in a Luer syringe, maintaining a 2 mm space short of the apex. The space was then filled with an MTA (Angelus Indústria de Produtos Odontológicos Ltda., Londrina, Paraná), plug, prepared by mixing the powder with saline solution. Prior to replantation, the alveolus was prepared by curettage using a surgical curette, irrigated with saline solution, and no stabilization was performed [11]. After replantation, all animals received a single dose of 20,000 IU of benzathine penicillin G (Fontoura Wyeth S.A.—São Paulo, São Paulo, Brazil) via intramuscular injection. The animals were housed in appropriately labeled cages and monitored until they fully recovered from anesthesia, before being returned to the animal facility.

### 2.3. Histological Processing

After 60 days, the animals were euthanized by an overdose of the anesthetic Vertanacol (Fort Dodge, IA, USA), containing 5% ketamine hydrochloride. The specimens corresponding to the right maxillae were immediately dissected and fixed in 10% formalin solution (Dinâmica, Indaiatuba, São Paulo, Brazil) (pH 7.4) for 24 h. As exclusion criteria, animals presenting suppuration in the periodontium of the replanted tooth, exfoliation of the replanted tooth, or fracture during the extraction and replantation procedures were excluded from the analysis.

The specimens were rinsed and subjected to decalcification in 4% EDTA solution (Êxodo Científica, São Paulo, São Paulo, Brazil) (pH 2.0). Subsequently, they were dehydrated in ascending concentrations of alcohol (Êxodo Científica, São Paulo, São Paulo, Brazil), cleared in xylene (Dinâmica, Indaiatuba, São Paulo, Brazil), embedded in low-melting-point paraffin (Inlab, São Paulo, São Paulo, Brazil) (56–58 °C) for 3 h, and included according to a pre-established and standardized orientation. Semi-serial longitudinal sections of the root, 6 µm thick, were obtained using an automatic rotary microtome Leica SMR 2000 (Leica Microsystems GmbH, Wetzlar, Hesse, Germany). In the sections mounted on glass slides (Olen, Ribeirão Preto, São Paulo, Brazil), paraffin was removed through a deparaffinization process using xylene (three xylene baths of 5, 10, and 15 min each), followed by rehydration in a descending alcohol series (90%, 70%, and 50%) and washing in phosphate-buffered saline (PBS) (Dinâmica, Indaiatuba, São Paulo, Brazil) 0.1 M, pH 7.4 (each wash lasting 10 min). After the deparaffinization and rehydration processes, the slides were immersed in hematoxylin solution (Prolab, São Paulo, São Paulo, Brazil) for 3 min and rinsed under running water. They were then immersed in eosin (Prolab, São Paulo, São Paulo, Brazil) for an additional 3 min, rinsed in distilled water, and dehydrated. Subsequently, after air-drying at room temperature, the slides were mounted using a mounting medium (Entellan, Merckmillipore, HE, Germany) and glass coverslips (Olen, Ribeirão Preto, São Paulo, Brazil). For histological analysis, the sections were examined under bright-field illumination using a standard light microscope (Leica—Aristoplan, Wetzlar, Hesse, Germany) connected to a digital camera Axiocam MRc (Carl Zeiss Microscopy GmbH, Jena, Thuringia, Germany). Visual fields of the analyzed structures from each animal were captured using 20× and 40× objective lenses with the AxioVision Rel. 4.0 software (Carl Zeiss GmbH, Oberkochen, Germany), adjusting brightness and contrast settings to enhance cellular and tissue visualization. Only the palatal surface of the root was considered, since rat teeth present a periodontal ligament exclusively on this surface. For histometric analysis, images of the slides from each animal were captured using a JVC digital camera (TK-1270, JVC, Yokohama, Kanagawa, Japan), attached to a Carl Zeiss microscope (Axiolab, Carl Zeiss, Oberkochen, Baden-Württemberg, Germany).

The descriptive analysis of the periodontal ligament, and the quantitative analysis of reattachment of periodontal ligament fibers, the presence of inflammatory resorption, replacement resorption, total resorption and ankylosis, were evaluated in relative percentage. The histological analysis was performed in a double-blind manner by trained researchers. 

These images, used for the collection of quantitative data, were processed and quantified using the ImageJ software (version 1.53t; National Institutes of Health, Bethesda, MD, USA). 

The qualitative analysis described the organization of periodontal ligament fibers, characterized by the perpendicular orientation to the root surface, and also the periodontal ligament thickness; the quantitative measurement of periodontal ligament reattachment were analyzed by the relative percentage of the total.

The total resorption, the percentage of inflammatory resorption, replacement resorption and ankylosis extension of the periodontal ligament was analyzed. Inflammatory resorption was considered when multinucleated cells were found adjacent to areas of dentin resorption, and an inflammatory infiltrate was present in the adjacent fibrous connective tissue. Replacement resorption was considered when the alveolar bone was in direct contact with the previously resorbed dentin, with or without inflammatory cells in the adjacent fibrous connective tissue. Ankylosis was considered when the periodontal ligament was replaced by alveolar bone tissue, which fused with the cementum layer. In these images, the total area of root dentin was first measured, followed by the area of resorbed root dentin. The values for the total dentin area and the resorbed dentin area were subjected to a rule-of-three calculation to quantify the percentage of the root affected by resorption. 

### 2.4. Statistical Analysis

The data were analyzed using the GraphPad Prism 3.0 software (GraphPad Software Incorporated, San Diego, CA, USA) with α = 0.05. After assessing the normality pattern of the sample using the Shapiro–Wilk test, the data were analyzed using the Kruskal–Wallis test and Dunn’s post hoc test for group comparisons, and were expressed as the mean ± standard deviation (SD).

## 3. Results

During the experimental period and histological processing one specimen was lost in the TR group (n = 9) due to root fracture, and two specimens were lost in the SE and IM groups (n = 8) due to suppuration in the periodontium of the replanted tooth. The total resorption area was used as the primary outcome variable, while ankylosis was considered the secondary outcome, for the purpose of power analysis. With a 5% margin of error, the statistical power was 85%. Weight gain among the animals showed no clinical differences between the experimental groups.

**Figure 1:**
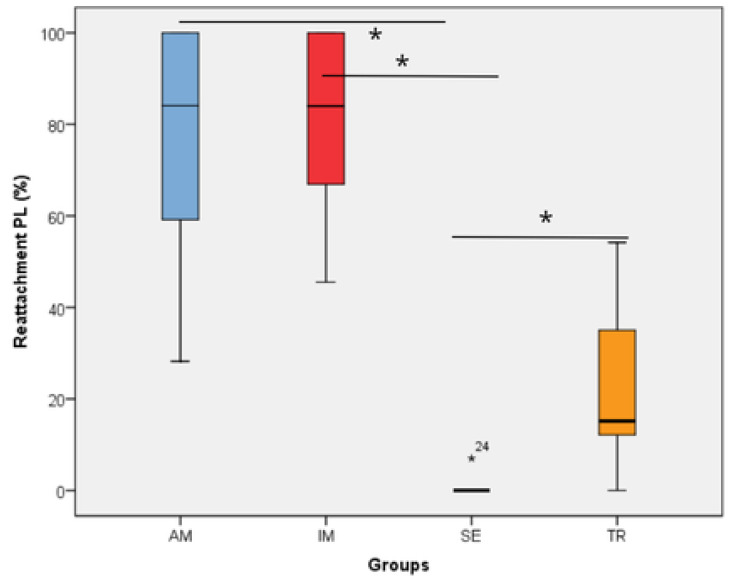
The reattachment periodontal ligament area in the experimental groups. * represents a statistically significant difference between groups. The statistical difference between groups was determined using the Kruskal–Wallis test and Dunn’s post hoc test. AM—ampicillin group, TR—tetracycline group, SE—dry medium group, IM—immediate replantation group. Black star represents outlier sample. Bold black stars (*) represent a statistically significant difference between groups.

**Figure 2:**
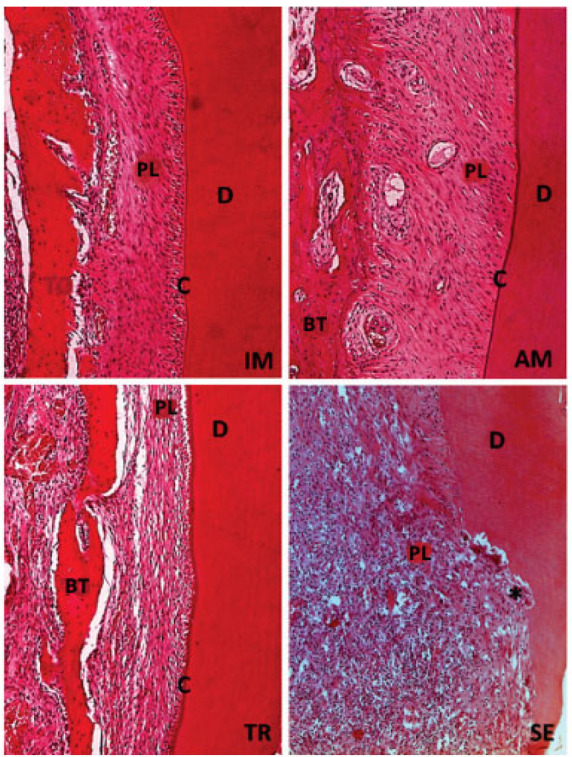
H&E staining. Original magnification 20×. The IM and AM groups show the presence of structured and organized periodontal ligament (PL), integrity of cementum layer (C), dentin (D), and bone tissue (BT). The TR group presents periodontal ligament (PL) without defined organization, but with intact cementum layer (C), dentin (D), and bone tissue (BT). The SE group the lack of organization in periodontal ligament (PL), with a large number of inflammatory cells, also a few areas with intact dentin (D) and bone tissue (BT), and absence of cementum can be seen; black star (*) represents an inflammatory resorption.

**Figure 3:**
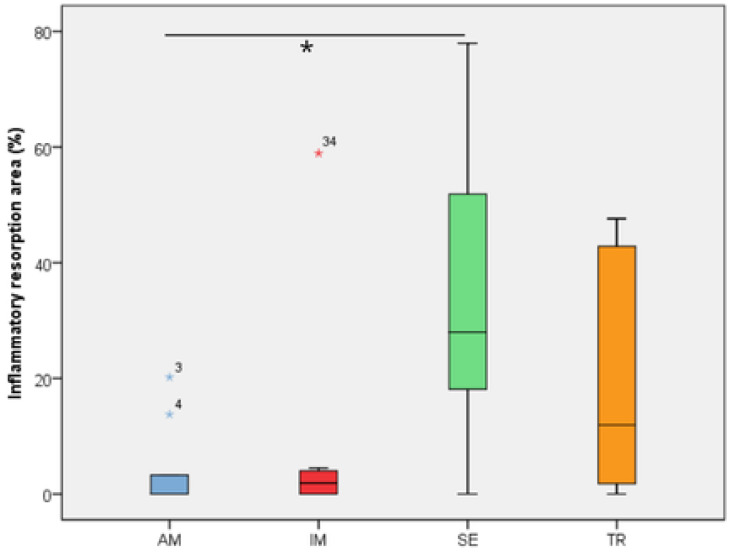
The inflammatory resorption area in the experimental groups. The statistical difference between groups was determined using the Kruskal–Wallis test and Dunn’s post hoc test. AM—ampicillin group, TR—tetracycline group, SE—dry medium group, IM—immediate replantation group. Blue and red stars represent outliers samples. * represents a statistically significant difference between groups.

**Figure 4:**
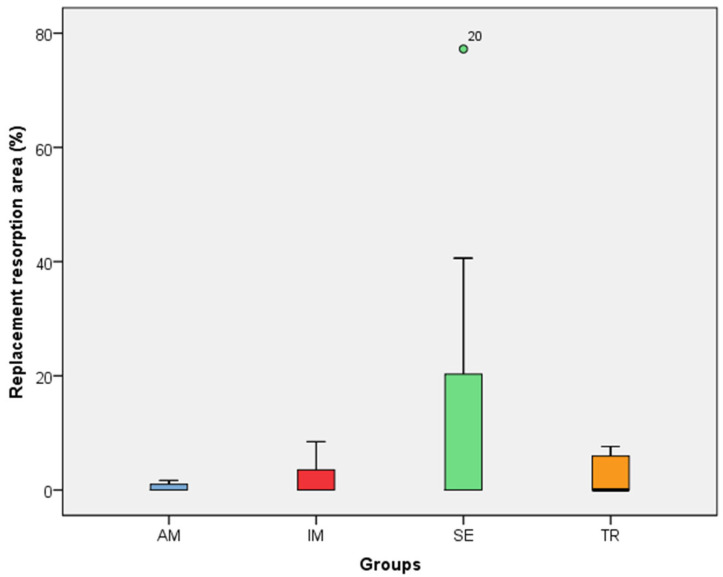
The replacement resorption area in the experimental groups. The statistical difference between groups was determined using the Kruskal–Wallis test and Dunn’s post hoc test. AM—ampicillin group, TR—tetracycline group, SE—dry medium group, IM—immediate replantation group. the green circle in the figure represents outlier sample.

**Figure 5:**
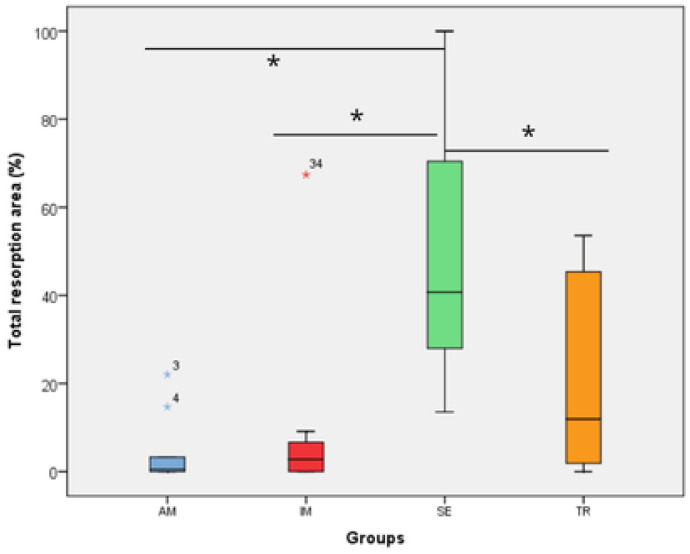
The total resorption area in the experimental groups. The statistical difference between groups was determined using the Kruskal–Wallis test and Dunn’s post hoc test. AM—ampicillin group, TR—tetracycline group, SE—dry medium group, IM—immediate replantation group. Blue and red stars represent outliers samples. * represents a statistically significant difference between groups.

**Figure 6:**
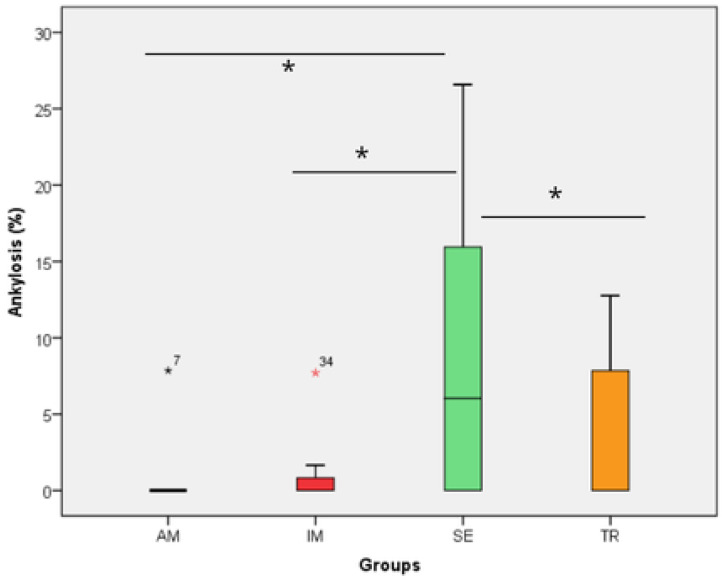
The ankylosis area in the experimental groups. The statistical difference between groups was determined using the Kruskal–Wallis test and Dunn’s post hoc test. AM—ampicillin group, TR—tetracycline group, SE—dry medium group, IM—immediate replantation group. Black and red stars represent outliers samples. Bold black stars (*) represent a statistically significant difference between groups.

**Figure 7:**
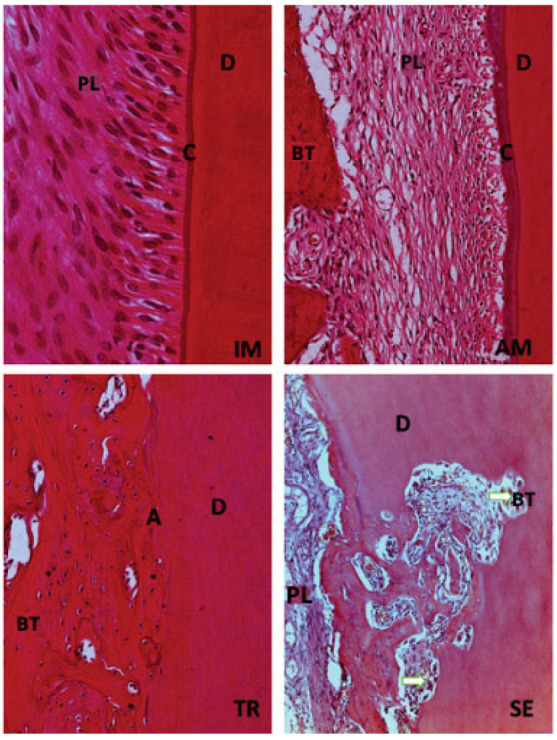
H&E staining. Original magnification 40×. Immediate replantation (IM) and Ampicillin (AM) groups show integrity of cementum layer (C) and dentin (D). The tetracycline (TR) group presents a larger percentage of ankylosis (A) observed in some specimens. Dry medium group (SE) is characterized by areas of inflammatory resorption (white arrow) found in several portions of the root.

All histomorphometric variables evaluated in each experimental group are presented in Figure 1, Figure 2, Figure 3, Figure 4, Figure 5, Figure 6 and Figure 7. In the analysis of the reattached periodontal ligament area, the SE group presented a significantly lower value compared to the IM, AM, and TR groups (IM group: *p* = 0.0002; AM group: *p* = 0.0002; TR group: *p* = 0.02) (Table 3 and Figure 1). The IM and AM groups exhibited a larger periodontal ligament thickness and a perpendicular fiber insertion pattern between the bone and cementum, whereas the TR and SE groups showed a smaller periodontal ligament thickness and greater disorganization of the periodontal ligament (Figure 2). The TR group displayed fibers arranged parallel to the bone and cementum, while the SE group showed completely disorganized fibers, with extensive inflammatory infiltrate and evidence of inflammatory root resorption (Figure 2).

In the analysis of the inflammatory resorption area, a significant difference was observed between the AM group, which showed the smallest percentage, and the SE group, that exhibited the largest one (*p* = 0.02) (Table 3, Figure 3). In the comparison of replacement resorption, no significant differences were observed among the groups (Table 3, Figure 4). With respect to the total resorption area, the SE group exhibited the highest values, showing significant differences when compared to the IM group (*p* = 0.005), AM group (*p* = 0.0007), and TR group (*p* = 0.03) (Table 3, Figure 5). The presence of ankylosis was higher in the SE group (IM group: *p* = 0.005; AM group: *p* = 0.0002; TR group: *p* = 0.03) (Table 3, Figure 6 and Figure 7).

## 4. Discussion

This study evaluated the influence of topical use of two antibiotics, tetracycline and ampicillin, on the repair process in the treatment of the root surfaces of teeth that underwent late replantation after being conditioned in whole milk for 60 min. The histological results found in the IM group demonstrate that the tooth extraction and replantation procedure did not interfere with the outcome, indicating the viability of the method. In this case, there was no impairment of the periodontal ligament’s vitality. Infection and the inflammatory response reduce the chances of periodontal ligament repair, a critical aspect for the survival of the replanted tooth [12,13]. In cases where periodontal repair was unfavorable, the presence of replacement or inflammatory resorption was observed.

When immediate replantation is not possible, the use of a preservation medium is recommended to prevent dehydration and preserve the vitality of the periodontal ligament [6,14]. The substance used must have an osmolarity, pH, and temperature compatible with the survival of the periodontal ligament. It should control inflammation, inhibit osteoclast activity, and be free from microbial contamination [7,15]. The healing process after replantation involves pulp and periodontal reactions that determine the prognosis of the replantation. In this case, endodontic treatment is indicated, with calcium hydroxide being the dressing of choice. This material has an effective action on bacteria and their toxins, which stimulate resorption related to infection. Since rat teeth have a large foramen, the use of an MTA plug allows the maintenance of the calcium hydroxide dressing inside the canal during the experimental period [16].

In an attempt to achieve the repair of these tissues in late-replanted teeth, research has been conducted evaluating the use of certain medications, such as fibroblast growth factor, 3Mix (a mixture of ciprofloxacin, metronidazole, and minocycline), and bisphosphonates, which have shown positive results in inhibiting osteoclastic activity, controlling inflammation, and regenerating the periodontal ligament [17,18,19]. Likewise, doxycycline and penicillin (amoxicillin) have also been studied with the same objective [6,7].

Contamination of the periodontal ligament may occur due to handling of the avulsed tooth, or even through contact with saliva from the oral cavity during the replantation procedure. The use of antibiotic substances has been developed with this possibility in mind, and tetracycline and its derivatives have shown promising results in both pulp and periodontal healing [12]. Minocycline, a derivative of tetracycline, has been associated with the suppression of osteoblastic differentiation and the acceleration of odontoblast-like cell differentiation in delayed tooth replantation [18]. In another study, the topical use of tetracycline reduced the incidence of ankylosis and supported the reintegration of the periodontal ligament, showing results comparable to those of immediate replantation [6]. According to the authors, this medication inhibits the activity of the metaloproteinases MMP-1 and MMP-8 [6]. The positive effect on periodontal repair may also be related to the excellent adsorption of tetracycline to the root surface, serving as a long-term reservoir for the drug [20].

Although tetracycline yielded positive results, it was not able to significantly reduce inflammatory resorption when compared to the group kept in dry storage. This finding aligns with a study where the treatment of late-replanted teeth with tetracycline showed greater inflammatory infiltrate compared to treatment with amoxicillin [9]. However, there are conflicting results regarding the use of this medication in replantation treatment. In a previous study, no beneficial effect on pulp revascularization and periodontal repair was observed when compared to teeth treated only with saline solution [21]. These differences may have arisen due to factors such as the animal model, the developmental stage of the replanted tooth, the extraoral time, the storage medium of the tooth, the replantation procedures, and the endodontic treatment [22].

The use of systemic antibiotic therapy with penicillin as part of the protocol is recommended by the International Association of Dental Traumatology (IADT) to help slow down the resorption process and reduce inflammation [9,10]. The use of penicillins is also inconsistent when compared to the use of tetracycline in the periodontal repair of replanted teeth. While some studies reveal that penicillin (amoxicillin) is superior to tetracycline [9,23], others show the opposite [24]. These findings may be due to the different dosages evaluated, which can affect the local concentration of the medication [7]. In this study, both medications were used topically and at the same concentration, and the AM group showed better results than the TR group, these results were comparable to those observed in the IM group, but significantly different from those in the SE group regarding inflammatory resorption, which was not observed in the TR group. Another factor that may have influenced the results between the two types of antibiotics tested is that the effect of tetracyclines is directly related to the concentration applied. At low concentrations, they can inhibit the activity of matrix metalloproteinases and collagenase, and their degradation of non-bony and bony connective tissues at normal concentrations has antibacterial activity by inhibiting bacterial protein synthesis [25].

These results are in accordance with other studies comparing the use of these same antibiotics after immediate and late replantation [9,23,26]. The differences in these findings may be explained by the fact that penicillins have a beta-lactam ring in their structure, with a broad-spectrum effect against Gram-positive bacteria and some Gram-negative bacteria found on the root of the avulsed tooth, which may have been contaminated prior to replantation [27]. Another factor that may have influenced the difference in results between the two types of antibiotics tested is that the effect of tetracyclines is directly related to the concentration applied. At low concentrations, they can inhibit the activity of matrix metalloproteinases and collagenase, and at normal concentrations, they have antibacterial activity by inhibiting bacterial protein synthesis [26]. However, at high concentrations, tetracyclines can cause tissue damage, probably due to their low pH around 3, which is lower than that of penicillins [28,29,30,31]. Therefore, since the pH of the environment where the root of the tooth is kept should be around 6–7 to maintain cell activity and growth [16], this likely contributed to penicillin having superior results in periodontal healing and inhibition of inflammatory resorption.

## 5. Conclusions

It is concluded that, in an aseptic environment, the topical use of antibiotics such as ampicillin and tetracycline in the treatment of the root surface in replantation of teeth stored in milk proves to be favorable to the process of periodontal ligament repair and reduction of root resorption. Notably, ampicillin demonstrated superior efficacy in mitigating inflammatory root resorption and better periodontal ligament reattachment.

## Figures and Tables

**Table 1:** Timeline for IM group.

Exodontia, endodontics, and replantation	Follow-up	
10 min	60 days	Euthanasia

**Table 2:** Timeline for AM, TR, and SE groups.

Exodontia, storage in milk or dry medium	Immersion in ampicillin, tetracycline, or saline solution	Endodontics and replantation	Follow-up	
60 min	5 min	10 min	60 days	Euthanasia

**Table 3:** The areas of resorption, periodontal ligament reinsertion, and ankylosis in the experimental groups.

Variables	Experimental Groups							
	AM		TR		SE		IM	
	Mean (SD)	Median	Mean (SD)	Median	Mean (SD)	Median	Mean (SD)	Median
Reattachment of PL (%)	77.9 (24.68) ^A^	84.08	22.57 (19.25) ^A^	15.19	0.87 (2.48) ^B^	0	80.92 (20.5) ^A^	84.01
Area of inflammatory resorption (%)	3.98 (7.12) ^A^	0	18.2 (20.86) ^AB^	11.94	34.24 (25.62) ^B^	27.98	8.84 (20.31) ^AB^	1.88
Area of replacement resorption(%)	0.41 (0.60) ^A^	0	2.28 (3.45) ^A^	0	14.73 (28.98) ^A^	0	1.93 (3.13) ^A^	0
Area of total resorption (%)	4.35 (7.66) ^A^	0.54	20.52 (22.31) ^A^	11.94	48.97 (29.08) ^B^	40.73	10.78 (23.08) ^A^	2.78
Ankylosis (%)	0 (0) ^A^	0	3.47 (5.35) ^A^	0	8.81 (10.44) ^B^	6.02	1.17 (2.70) ^A^	0

The statistical differences between groups were determined using the Kruskal–Wallis test and Dunn’s post hoc test. AM—ampicillin group, TR—tetracycline group, SE—dry medium group, IM—immediate replantation group, SD—standard deviation. Different letters indicate a statistically significant difference between groups for each event.

## Data Availability

The original contributions presented in the study are included in the article, further inquiries can be directed to the corresponding author.
